# State-dependent modulation of positive and negative affective valences by a parabrachial nucleus-to-ventral tegmental area pathway in mice

**DOI:** 10.3389/fncir.2023.1273322

**Published:** 2023-11-29

**Authors:** Takashi Nagashima, Kaori Mikami, Suguru Tohyama, Ayumu Konno, Hirokazu Hirai, Ayako M. Watabe

**Affiliations:** ^1^Institute of Clinical Medicine and Research, Research Center for Medical Sciences, The Jikei University School of Medicine, Chiba, Japan; ^2^Gunma University Graduate School of Medicine, Maebashi, Japan; ^3^Viral Vector Core, Gunma University Initiative for Advanced Research (GIAR), Maebashi, Japan

**Keywords:** parabrachial nucleus, ventral tegmental area, glutamic acid decarboxylase 65, avoidance, aversive memory, operant task, optogenetics, mice

## Abstract

Appropriately responding to various sensory signals in the environment is essential for animal survival. Accordingly, animal behaviors are closely related to external and internal states, which include the positive and negative emotional values of sensory signals triggered by environmental factors. While the lateral parabrachial nucleus (LPB) plays a key role in nociception and supports negative valences, it also transmits signals including positive valences. However, the downstream neuronal mechanisms of positive and negative valences have not been fully explored. In the present study, we investigated the ventral tegmental area (VTA) as a projection target for LPB neurons. Optogenetic activation of LPB-VTA terminals in male mice elicits positive reinforcement in an operant task and induces both avoidance and attraction in a place-conditioning task. Inhibition of glutamic acid decarboxylase (GAD) 65-expressing cells in the VTA promotes avoidance behavior induced by photoactivation of the LPB-VTA pathway. These findings indicate that the LPB-VTA pathway is one of the LPB outputs for the transmission of positive and negative valence signals, at least in part, with GABAergic modification in VTA.

## Introduction

The state-dependent regulation of adaptive behavior is critical for animal survival. Sensory information such as pain and taste signals arising from peripheral tissues is transmitted to the lateral parabrachial nucleus (LPB) in the pons directly via the dorsal horn of the spinal cord and indirectly via the nucleus of the solitary tract (NTS) through the vagal ganglia (Chiang et al., 2020; Tan et al., 2020). Increasing evidence suggests that LPB plays a crucial role in nociceptive and aversive signal processing (Watabe et al., 2013; Palmiter, 2018; Chiang et al., 2019; Nagase et al., 2019). The LPB neurons, including those expressing calcitonin gene-related peptide (CGRP), play a key role in transmitting aversive signals and are involved in avoidance behaviors, fear learning, taste aversion, and appetite suppression (Carter et al., 2013; Han et al., 2015; Chen et al., 2018; Bowen et al., 2020). In contrast, SatB2 neurons in the PB exhibit responses to sweet tastes, with certain SatB2 neurons encoding a positive valence that induces appetitive lick behavior (Fu et al., 2019). Nevertheless, the downstream circuit mechanism of LPB for negative and positive valences remains largely unexplored.

The LPB transmits information to multiple downstream targets, such as the ventral tegmental area (VTA), bed nucleus of the stria terminalis (BNST), central amygdala (CeA), insular cortex (IC), lateral hypothalamus (LH), periaqueductal gray (PAG), parasubthalamic nucleus (PSTN), substantia innominata (SI), ventromedial hypothalamus (VMH), and ventral posteromedial thalamic nucleus (VPMpc) (Chiang et al., 2019; Bowen et al., 2020; Yang et al., 2021; Nagashima et al., 2022). VTA, a projection target of the LPB, is a heterogeneous structure comprising dopamine, glutamate, and inhibitory neurons, and plays a critical role in motivated behavior, learning, and psychiatric disorders (Morales and Margolis, 2017). Whole-brain mapping with retrograde tracers showed projections from the PB region to the VTA inhibitory neurons (Beier et al., 2015). Other histological and electrophysiological analyses have indicated projections from the LPB to the VTA dopamine neurons (Yang et al., 2021). Furthermore, sweet and bitter taste stimuli activate LPB neurons projecting to the VTA (Boughter et al., 2019), suggesting that both appetitive and aversive information is transmitted by the LPB-VTA pathway.

Previous studies have indicated that the LPB-VTA pathway is involved in signals associated with positive valences (Boughter et al., 2019; Yang et al., 2021), whereas the LPB transmits signals associated with negative valences (Palmiter, 2018; Nagase et al., 2019). However, the circuit mechanisms of these opposing valences in behavioral regulation have not been fully explored. Therefore, to examine how the LPB-VTA pathway controls opposing emotional behaviors, we performed optogenetic and pharmacogenetic experiments. The results suggest that the LPB-VTA contributes to avoidance/attraction behaviors and that VTA GAD65 cells are involved in inhibiting avoidance behavior.

## Materials and methods

### Animals

All the experimental protocols in this study including the use of animals were approved by the Institutional Animal Care and Use Committee of the Jikei University (Tokyo, Japan; Approval No. 2018-030 and 2019-010). All experiments were conducted in accordance with the guidelines described in the Guidelines for Proper Conduct of Animal Experiments by the Science Council of Japan (2006) and those recommended by the International Association for the Study of Pain. Every effort was made to reduce the number of animals used and their suffering during the procedure. Male C57BL/6J mice (Japan SLC, Shizuoka, Japan) were group-housed under a 12 h light/dark cycle. Food and water were available *ad libitum*.

### Adeno-associated virus

AAV1-hSyn-Chronos:GFP (purchased from UNC Vector Core), AAVDJ-hSyn-EYFP (a generous gift from Prof. Toshihisa Ohtsuka, University of Yamanashi), AAV1-mGAD65-Cre, AAV1-hSyn-FLEX-Chronos:GFP (purchased from UNC Vector Core), and AAVDJ-EF1a-FLEX-hM4Di-2A-cgfTagRFP (a generous gift from Prof. Kazuto Kobayashi, Fukushima Medical University) were used for microinjection.

### Stereotaxic surgery

Male mice (4–5 weeks) were intraperitoneally anesthetized with a mixture of medetomidine hydrochloride (0.75 mg/kg; Zenoaq, Fukushima, Japan), midazolam (4.0 mg/kg; Astellas, Tokyo, Japan), and butorphanol tartrate (5.0 mg/kg; Meiji Seika Pharma, Tokyo, Japan). Bilateral stereotaxic microinjections of adeno-associated virus (AAV) (0.25 μL) into the LPB (6.0 mm posterior to bregma, 1.5 mm lateral to midline, and 3.4 mm ventral to the skull surface, with a 20° anterior-to-posterior angle to avoid damaging the superficial arteries during surgery) and AAV (0.30 μL) into the VTA (3.4 mm posterior to bregma, 0.7 mm lateral to midline, and 4.3 mm ventral to the skull surface) were performed using a Hamilton microsyringe (1701RN Neuros Syringe, 33 G, 10 μL; Hamilton Company, Reno, NV, United States). The microinjection speed (50 nL/min) was controlled by a microsyringe pump (UMP3; UltraMicroPumpII with SYS-Micro4 Controller, UMP2, UMC4; World Precision Instruments, Sarasota, FL, USA). After 3–5 weeks, a second surgical procedure was performed for the placement of a bilateral light-emitting diode (LED) cannula unit consisting of dual optical fibers (0.25 mm in diameter, 4.0 mm in length, and 1.1 mm in spacing) attached to a LED body (blue, 470 nm) (TeleLCD-B-4.0-250-1.1; Bio Research Center, Tokyo, Japan). The LED cannula unit was stereotactically implanted bilaterally above the VTA (3.3 mm posterior to bregma), and fixed to the skull with dental cement (GC Fuji I; GC Corporation, Tokyo, Japan). Mice were allowed to recover for several days.

### Lever-press task

Mice were placed in an operant task chamber (OPR-3002; O’Hara & Co., Ltd.) and trained to perform lever presses to obtain small sucrose reward pellets (1811213, TestDiet), as previously described (Nagashima et al., 2022). Mice were deprived of food for 18–24 h before the training sessions and allowed access to conventional food after the training sessions. The training procedure consisted of a 1 day magazine training and 2–3 days operant training. In the 15 min magazine training, the mice were randomly presented with a food pellet immediately after a tone. In the 30 min operant training, mice were presented with a food pellet immediately after a tone when they pressed a lever. Only mice that pressed the lever at least 20 times during the 30 min training were selected for subsequent experiments. Optogenetic manipulation was performed after the training sessions with rewarding food pellets, wherein LED stimulation was presented instead of food pellets. Teleopto-receiver (2 g, TeleR-2-P, an infrared-driven wireless LED unit; Bio Research Center) was attached immediately before the test. The mice received 5 ms of LED illumination without food pellets or tones when they pressed a lever. The Operant Task Studio V2 software (O’Hara & Co., Ltd., Tokyo, Japan) was used to measure the timing of the lever presses.

In experiments involving optogenetic manipulation, we first investigated the relationship between the number of light pulses at 40 Hz and lever-press behavior. The 10 min sessions were validated in the following order: 1 pulse, 4 pulses, and 40 pulses. The next day, we further examined the relationship between the frequency of light stimuli per second and lever-press behavior. The 10 min sessions were validated in the following order: 4 Hz, 20 Hz, and 40 Hz. On the final day, the mice received 10 pulses at 20 Hz when they pressed a lever during a 20 min test session.

### Real-time Y-maze test

This place conditioning task was conducted in a custom-made Y-shaped maze apparatus (YM-3002; O’Hara & Co., Ltd., Tokyo, Japan) placed in a sound-attenuating chamber (CL-M3; O’Hara & Co., Ltd.), as described previously (Ito et al., 2021; Nagashima et al., 2022). The Y-maze consists of three arms with different textures (Arm 1, punched metal; Arm 2, grid metal; Arm 3, mesh metal). Mice were allowed to habituate for 10 min to the Y-maze apparatus (10 lux, 50 dB background white noise), in which a divider was inserted in Arm 1 so that a mouse could only freely move between Arm 2 and Arm 3. More than 2 h after the habituation session, the conditioning session was conducted for 10 min. Teleopto-receiver (2 g, TeleR-2-P, an infrared-driven wireless LED unit; Bio Research Center) was attached to the LED cannula unit immediately before the conditioning session. Optical stimulation was controlled using the Time OFCR1 software (O’Hara & Co., Ltd.) and a programmable stimulator (Master-8; A.M.P. Instruments Ltd., Jerusalem, Israel); specifically, whenever the mouse entered the LED area in Arm 2, it received 5 ms of optical stimulation (40 Hz) that was controlled by an infrared-driven remote controller (Teleopto remote controller; Bio Research Center). For comparison, a non-stimulated control area (no-LED area) of the same size as the LED area was designed on the opposite side in Arm 3. The next day, a 10 min retrieval session was performed in the same manner as the conditioning session, while no optical stimulation was applied.

Mouse behavior was captured at 2 frames per second. Time spent in each LED and no-LED area, and the number of each area entry was analyzed by Time OFCR1 software. Moving speed for 3 s after leaving the LED area was analyzed based on the coordinates of the center of gravity. Trials that returned to the LED area within 3 s were excluded. Mice that did not exit the LED area were also excluded.

In chemogenetic inhibition of GAD65 cells throughout the VTA during stimulation of the LPB-VTA pathway, the real-time Y-maze task was performed for 2 days. Different floor contexts were used for day 1 (Arm 1, punched metal; Arm 2, grid metal; Arm 3, mesh metal) and day 2 (Arm 1, grid metal; Arm 2, mesh metal; Arm 3, punched metal). More than 2 h after the 10 min habituation session, the 10 min conditioning session was conducted. Administration of saline or 1 mg/kg clozapine N-oxide (CNO) was alternated for each mouse 30 min before the conditioning session.

### Histological observation

To confirm expression and cannula location in mice injected with AAV was observed using a fluorescence microscopy (BZ-X710, Keyence). The cannula position of two specimens in YFP mice was excluded because of the indistinct tip. Fluorescent images of the VTA GAD65 cells receiving input from LPB neurons were acquired using a confocal microscope (FV3000, Olympus). Coronal sections (100 μm) were mounted with DAPI-containing encapsulated material (P36966, Invitrogen). Each brain region was identified using the Allen Brain Reference Atlas^1^ (Lein et al., 2007). The images were minimally processed using Fiji (Schindelin et al., 2012) to contrast for optimal representation of the data.

### Statistical analysis and graph creation

All data were analyzed using GraphPad Prism 9 software (GraphPad Software, La Jolla, CA). A *p*-value less than 0.05 was considered a significant difference. Graphs were created using GraphPad Prism, the Python software, and Biorender.com.

## Results

### Optogenetic activation of the LPB-VTA pathway promoted positive reinforcement

**Figure 1:**
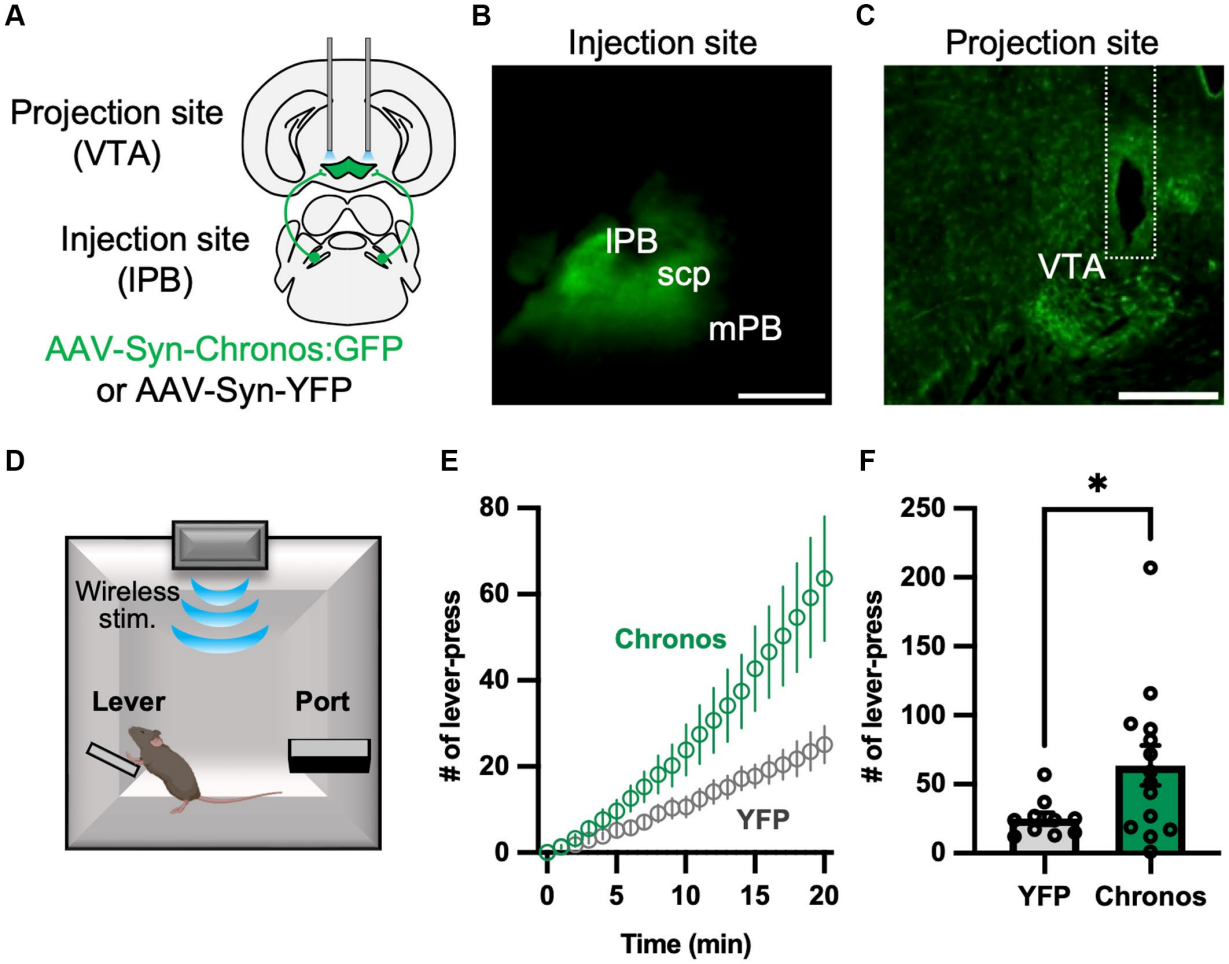
Photoactivation of the LPB-VTA pathway in the lever-press paradigm. **(A)** Schematic representation of the microinjection and placement of the LED cannula unit. **(B,C)** Images of the injection site **(B)** and projection site **(C)**. The dashed line indicates the position of the optical fiber. The scale bars represent 0.5 mm. scp, superior cerebellar peduncle. **(D)** Schematic of the lever press task. **(E)** Time-series plots of the number of lever-press responses (YFP, *n* = 10; Chronos, *n* = 14). **(F)** Summary of the number of lever-press responses (YFP, *n* = 10; Chronos, *n* = 14). ^*^p < 0.05 (unpaired two-sided *t*-test). Data are presented as mean ± SEM.

We recently confirmed that LPB neurons project to the VTA (Nagashima et al., 2022). Previous studies demonstrated that VTA dopaminergic neurons promote lever-press behavior during operant tasks (Adamantidis et al., 2011; Saunders et al., 2018). To elucidate the function of the LPB-VTA pathway, we conducted a lever-press task combined with optogenetic stimulation. To achieve optogenetic activation of axonal terminals, we conducted bilateral AAV injections (AAV-Syn-Chronos:GFP or AAV-Syn-YFP) into the LPB to express the channelrhodopsin (Chronos) or YFP and placed dual optic cannulae over the VTA (Figures 1A–C; Supplementary Figure S1A). During the shaping period, mice were primarily trained to press a lever using a real food pellet prior to optogenetic stimulation. Through optogenetic manipulation, we systematically validated the impact of the number of light pulses and stimulus frequency after the food reward training. Notably, we observed a significant difference in the number of lever presses between the YFP-control and Chronos mice during stimulation at 20 Hz (Supplementary Figures S1B–G). Subsequently, they were subjected to a test in which LED illumination (5 ms, 10 20 Hz pulses) was delivered only when they pressed a lever (Figure 1D). Chronos mice exhibited a higher number of lever presses compared to YFP mice (Figures 1E,F). These results suggest that the LPB-VTA pathway serves as a signal that promotes positive reinforcement in the lever-pressing behavioral paradigm.

### Activation of the LPB-VTA pathway induces avoidance and attraction behavior

**Figure 2:**
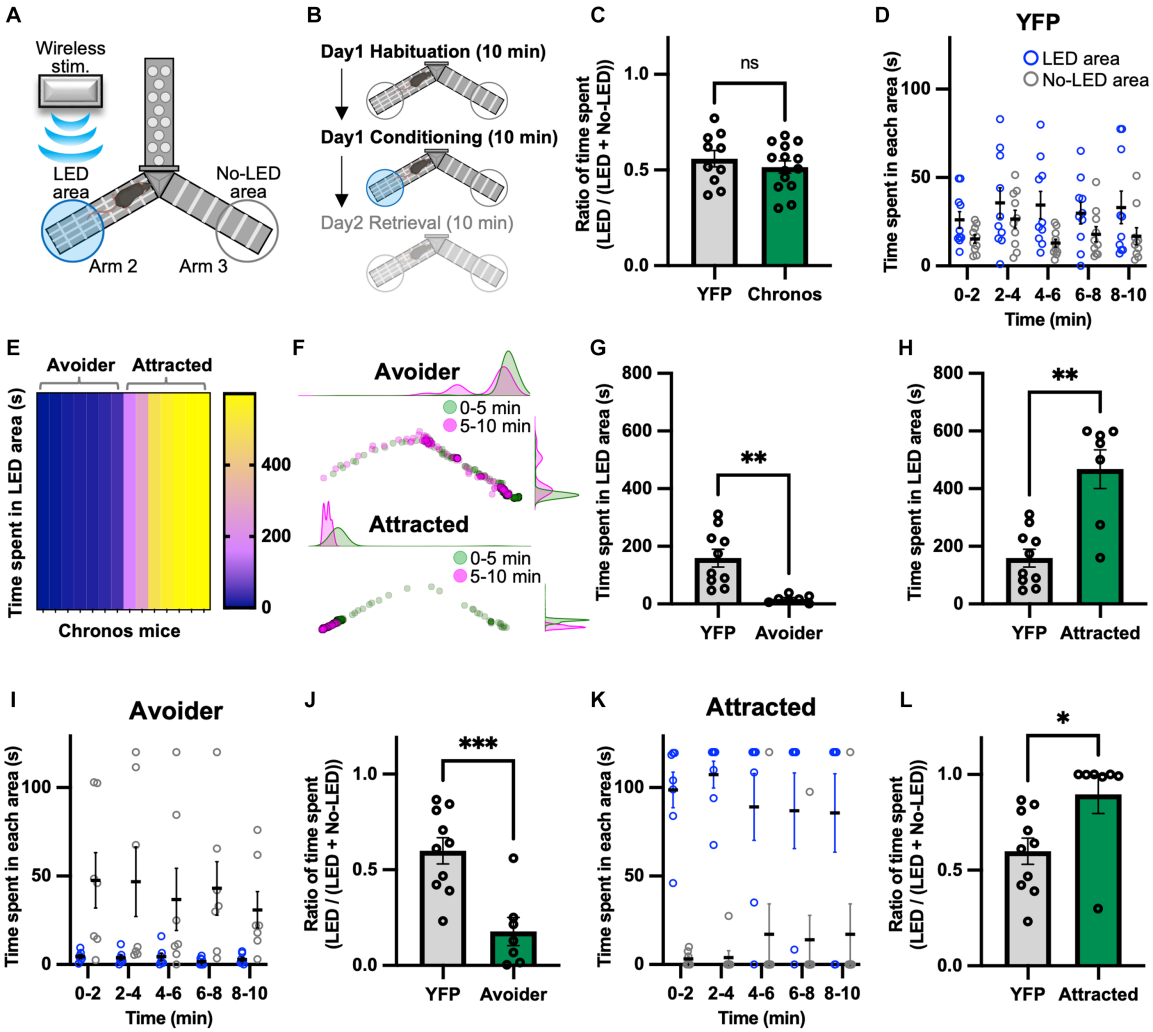
Photoactivation of the LPB-VTA pathway in the real-time place avoidance paradigm. **(A)** Schematic illustrations of the real-time place avoidance test using a Y-shaped apparatus. **(B)** Experimental schedule of the test. **(C)** Summary of the ratio of time spent during the 10 min habituation session (YFP, *n* = 10; Chronos, *n* = 14). **(D)** Time spent in each area during each 2 min interval (YFP, *n* = 10). **(E)** The heatmap represents time spent in the LED area in the 10 min conditioning session for individual mice (Chronos, *n* = 14). **(F)** Density plots and a scatter plot showing the position of a representative avoider (top) and attracted (bottom) mouse every 0.5 s in the 10 min conditioning session. **(G,H)** Time spent in the LED area in the 10 min conditioning session (YFP, *n* = 10; avoider, *n* = 7; attracted, *n* = 7). **(I–L)** Time spent in each area during each 2 min interval **(I,K)** and summary of the ratio of time spent during the 10 min conditioning session **(J,L)**. ns, *p* > 0.05; ^*^p < 0.05, ^**^p < 0.01, and ^***^p < 0.001 (unpaired two-sided *t*-test). Data are presented as mean ± SEM.

A real-time Y-maze test was performed to verify the emotional value of the signal generated by photoactivating the LPB-VTA pathway using the same mice used for the lever-press task. Previously, we assessed real-time place-avoidance behavior using a Y-shaped apparatus with a wireless photostimulation system (Figure 2A) (Ito et al., 2021; Nagashima et al., 2022). During the habituation session, mice were allowed to explore the apparatus for 10 min without photostimulation (Figure 2B). There was no significant difference in the ratio of time spent [LED area/(LED area + no-LED area)] in the habituation session (Figure 2C).

The conditioning session was conducted more than 2 h after the habituation session. The mice freely explored the apparatus between Arms 2 and 3 and received photostimulation (5 ms, 40 Hz) whenever they entered the LED area. We chose the 40 Hz stimulation in the present study because we have previously used the same apparatus to investigate downstream circuits of the LPB under the same stimulus conditions (Ito et al., 2021; Nagashima et al., 2022). We confirmed that the YFP mice showed no significant bias in the time spent in the two areas (Figure 2D). Surprisingly, among Chronos mice, there were two distinct groups: those that avoided the LED area and those that approached the LED area (Figures 2E,F). Hence, the Chronos mice were classified as avoider or attracted groups based on the mean time spent in the LED area in YFP mice. The time spent in the LED area was lower for avoider mice than for YFP, whereas it was significantly higher for attracted mice (Figures 2G,H). Avoider mice stayed in the no-LED area rather than in the LED area at any point in each 2 min interval and showed a lower ratio of time spent during the 10 min conditioning session (Figures 2I,J). In contrast, attracted mice stayed in the LED area rather than in the no-LED area and exhibited a higher ratio of time spent there (Figures 2K,L).

We have previously demonstrated that activation of the LPB-PSTN pathway induces escape behavior (Nagashima et al., 2022). Glutamate neurons in the VTA also play a role in escape responses to threatening stimuli (Barbano et al., 2020). Therefore, we determined whether the LPB-VTA pathway induces escape behavior. The moving speed after leaving the LED area was analyzed as a proxy for escape behavior (Supplementary Figure S2A), as previously reported (Nagashima et al., 2022). Moving speed for 3 s after leaving the LED area was analyzed at 0.5 s intervals and visualized as a heatmap, where bright colors indicate more rapid movement (Supplementary Figures S2B,C,E). Although some individuals exhibited higher moving speeds 1 s after leaving the LED area, no significant difference was observed between avoider and YFP mice (Supplementary Figure S2D). In contrast, a decrease in moving speed 1 s after leaving the LED area was observed in attracted mice (Supplementary Figure S2F). This may be due to the large number of attracted mice approaching the LED area.

Recently, Malenka’s groups demonstrated that terminal activation of the amygdala-nigra pathway elicited reinforcement when linked to voluntary actions, but failed to support Pavlovian associations (Steinberg et al., 2020). To understand the relationship between voluntary lever-press behavior and avoidance behavior during place conditioning in the same individual mice, we analyzed lever-press frequency of both avoider and attracted mice compared to YFP mice (Supplementary Figure S3A). We further explored the correlation between avoidance behavior and number of lever presses by calculating Pearson’s correlation coefficients (Supplementary Figures S3B,C). Avoider mice that exhibited weaker avoidance had an increased number of lever presses, but no significant correlation was detected between avoider (*r* = 0.53, *p* = 0.22) and attracted mice (*r* = 0.32, *p* = 0.48). Thus, our results suggest that signals that induce avoidance behavior in place conditioning can promote spontaneous lever-pressing behavior.

### Activation of the LPB-VTA pathway forms aversive memory

**Figure 3:**
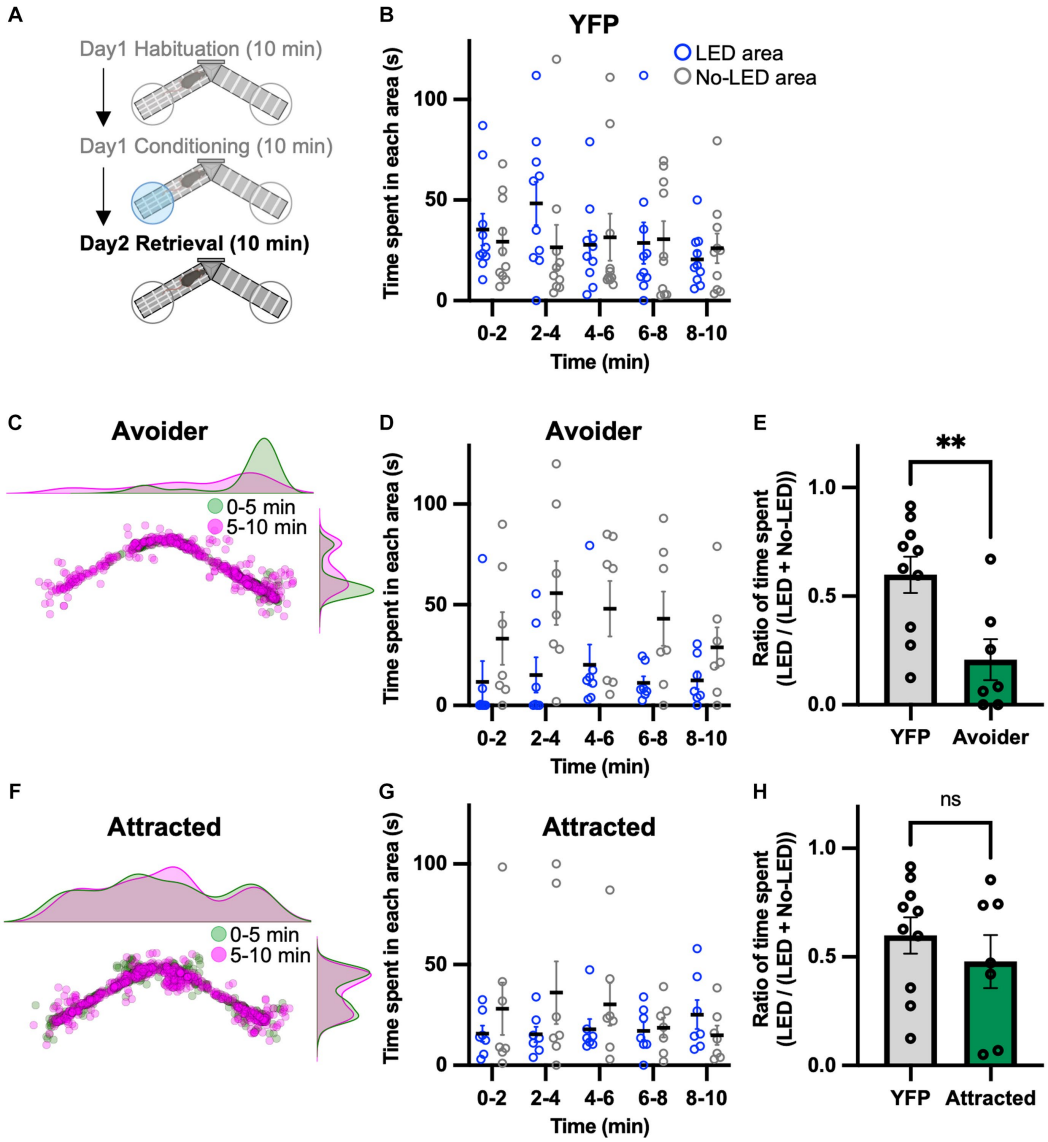
Aversive memory is detected in the retrieval session. **(A)** Experimental schedule of the test. **(B)** Time spent in each area during each 2 min interval (YFP, *n* = 10). **(C)** Density plots and a scatter plot showing the position of a representative avoider mouse every 0.5 s during the 10 min retrieval session. **(D)** Time spent in each area during each 2 min interval (avoider, *n* = 7). **(E)** Summary of ratios of time spent for 0–5 min (YFP, *n* = 10; avoider, *n* = 7). **(F)** Density plots and a scatter plot showing the position of a representative attracted mouse every 0.5 s during the 10 min retrieval session. **(G)** Time spent in each area during each 2 min interval (attracted, *n* = 7). **(H)** Summary of ratios of time spent for 0–5 min (YFP, *n* = 10; attracted, *n* = 7). ns, *p* > 0.05 and ^**^p < 0.01 (unpaired two-sided *t*-test). Data are presented as mean ± SEM.

Because of the seemingly contradictory results, we conducted a place memory task using LPB-VTA photostimulation as an unconditioned stimulus to directly examine whether this pathway acts as a negative or positive affective valence. To investigate the effect of LPB-VTA pathway activation on memory formation, a memory retrieval test was performed the day after the conditioning session (Figure 3A). The mice were tested using the same apparatus used for the conditioning session but without photostimulation. YFP mice showed no significant bias in the time spent in these two areas (Figure 3B). Chronos mice were analyzed in the same groups (avoider and attracted) as in the conditioning session. Avoider mice stayed in the no-LED area longer than in the LED area (Figures 3C,D). In particular, the ratio of the time spent [LED area/(LED area + no-LED area)] from 0 to 5 min was significantly shorter in avoider mice (Figure 3E). In contrast, there was no significant bias in the time spent in either area by the attracted mice (Figures 3F–H). These results support the notion that avoidance behavior induced by photoactivation of the LPB-VTA pathway forms an aversive memory.

### LPB neurons project to the GAD65 cells in the VTA

**Figure 4:**
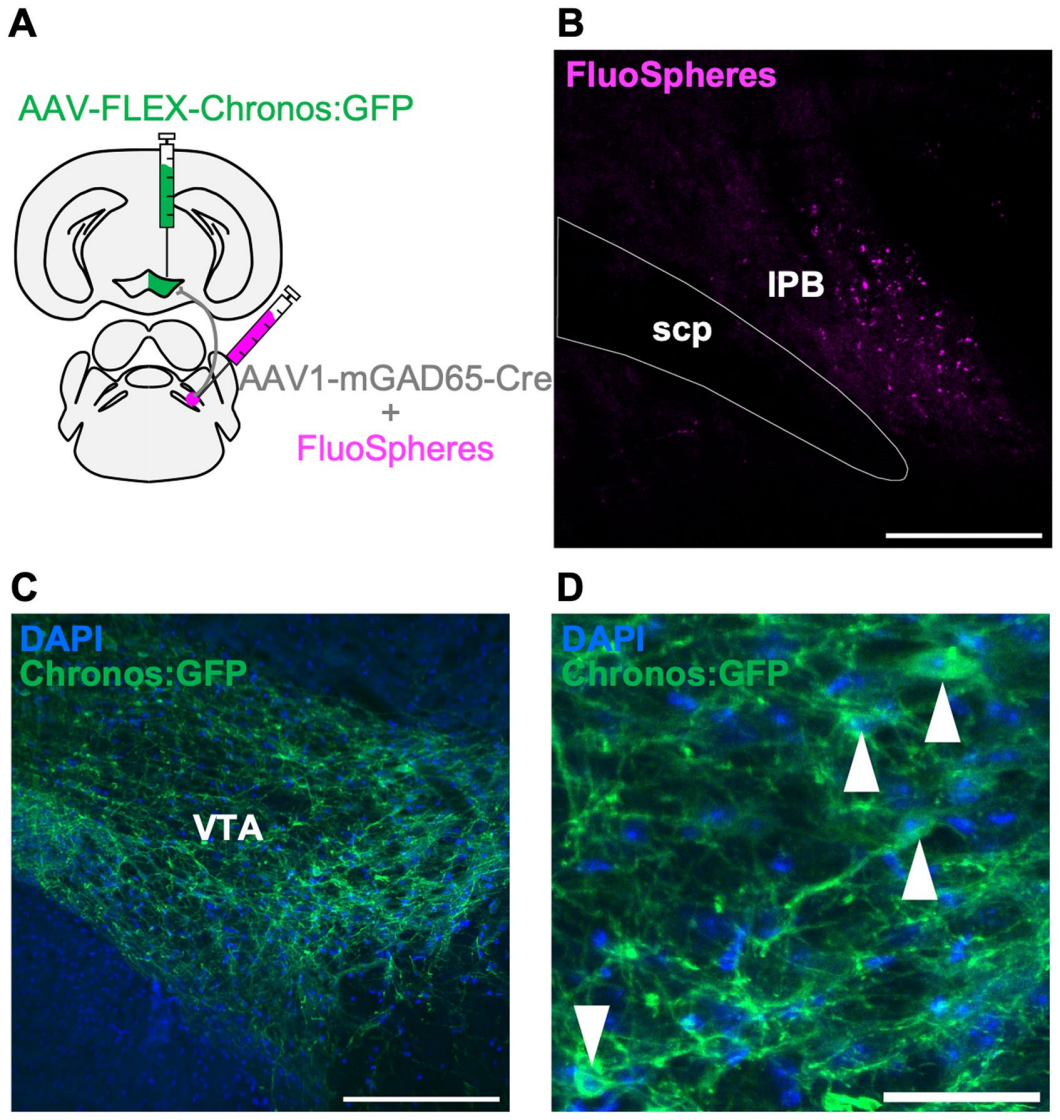
LPB neurons project to the GAD65 cells in the VTA. **(A)** Schematic of microinjection. **(B–D)** Representative images of fluorescent microspheres (FluoSpheres) at the injection site **(B)** and Chronos:GFP fluorescence (green) merged with DAPI (blue) at the projection site **(C,D)**. Arrowheads point to the cell bodies of Chronos:GFP-expressing cells. Scale bars represent 200 μm **(B,C)** or 50 μm **(D)**. scp, superior cerebellar peduncle.

To further characterize the LPB-VTA pathway, we histologically examined the postsynaptic cell types. The VTA is a heterogeneous structure comprising dopamine, glutamate, and inhibitory neurons (Morales and Margolis, 2017). The circuit from LPB neurons to VTA dopamine neurons has been reported to play a role in place preference behavior in mice (Yang et al., 2021). Whole-brain connectivity analysis demonstrated that neurons in the pons project to excitatory and inhibitory neurons in the VTA (An et al., 2021). Histological experiments with retrograde tracers showed projections from the PB region to the VTA inhibitory neurons (Beier et al., 2015). However, the circuit from the LPB to the VTA-inhibitory cells is not fully understood. In this study, we used GAD65, a marker molecule of inhibitory cells, to investigate the presence of a circuit from the LPB to GAD65-expressing cells in the VTA, using a histological approach. A previous study demonstrated that the expression of Cre recombinase gene carried by AAV serotype 1 (AAV1-Cre) can exhibit anterograde trans-synaptic spread property (Zingg et al., 2017). In this study, we unilaterally injected AAV1-mGAD65-Cre, which expresses Cre specifically in inhibitory cells by the mGAD65 promoter (Hoshino et al., 2021), together with fluorescent microspheres (FluoSpheres) into the LPB and AAV-Syn-FLEX-Chronos:GFP into the VTA of the same individuals (Figures 4A,B). This approach allowed us to characterize the projection from the LPB to VTA inhibitory cells. Notably, we observed GFP fluorescence in the VTA cells (Figures 4C,D), indicating the presence of a circuit from LPB neurons to GAD65 cells in the VTA.

### Inhibition of GAD65 cells in the VTA promotes avoidance behavior induced by activation of the LPB-VTA pathway

**Figure 5:**
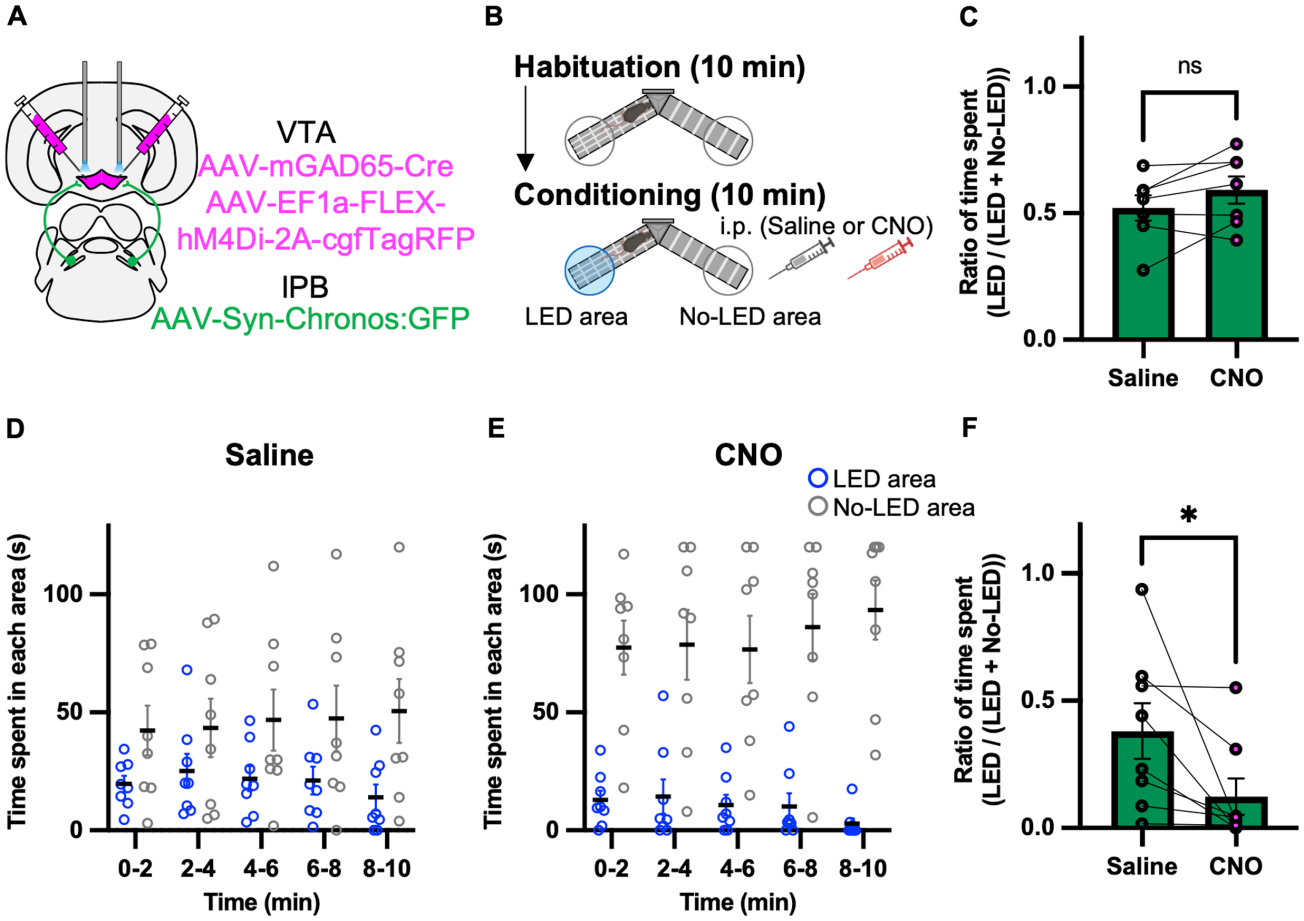
Inhibition of the GAD65 cells in VTA promotes avoidance behavior. **(A)** Schematic of microinjection. **(B)** Experimental schedule of the test. **(C)** Summary of the ratio of time spent in the 10 min habituation session (Saline, *n* = 7; CNO, *n* = 7). **(D,E)** Time spent in each area during each 2 min interval of the Saline group **(D)** or the CNO group **(E)**. **(F)** Summary of the ratio of time spent for 5–10 min of the conditioning session (Saline, *n* = 8; CNO, *n* = 8). ns, *p* > 0.05 and ^*^p < 0.05 (paired two-sided *t*-test). Data are presented as mean ± SEM.

We hypothesized that the GABAergic inhibitory neurons in the VTA play a role in the behavioral effects of the LPB-VTA pathway. To assess the physiological relevance of GAD65 cells in the LPB-VTA pathway at the behavioral level, we performed optogenetic approach combined with pharmacogenetic inhibition of GAD65 cells throughout the VTA using inhibitory designer receptors exclusively activated by designer drugs (DREADD) that were designed based on the human M4 muscarinic receptor. AAV-Syn-Chronos:GFP was injected into the bilateral LPBs, and AAV1-mGAD65-Cre and AAV-EF1a-FLEX-hM4Di-2A-cgfTagRFP were injected into the bilateral VTAs (Figure 5A). Dual optic cannulae were implanted over the VTA for optogenetic activation of axonal terminals. A real-time place-avoidance test was performed using the apparatus as in Figure 2 (Figure 5B). Mice were habituated to the apparatus, and no significant difference was detected in the ratio of time spent [LED area/(LED area + no-LED area)] during the 10 min habituation session (Figure 5C). The conditioning session was conducted more than 2 h after the habituation session. For the pharmacogenetic manipulation, clozapine N-oxide (CNO) or saline was injected 30 min before the conditioning session. No significant effect of CNO on locomotor activity was observed (Supplementary Figure S4A). CNO administration decreased the time spent in the LED area and remarkably increased the time spent in the no-LED area during each 2 min interval (Figures 5D,E). The ratio of time spent [LED area/(LED area + no-LED area)] in the 5–10 min conditioning session was lower in all mice and was significantly decreased by CNO administration (Figure 5F). These results suggest that GAD65 cells in the VTA are involved in inhibiting avoidance behavior induced by the activation of the LPB-VTA pathway.

## Discussion

In the present study, we conducted photoactivation of axon terminals in the LPB-VTA pathway and provided evidence for its role in positive reinforcement using an operant task and in avoidance/attraction behaviors using the real-time Y-maze paradigm. Despite extensive research on neural circuits involving the VTA dopamine neurons, the projection from LPB neurons to VTA neurons and their functions remain largely unknown. The LPB receives aversive signals and transmits them to several brain regions (Chiang et al., 2019; Bowen et al., 2020). The present study sheds light on the neuronal mechanisms of the positive and negative affective signals from the LPB to the VTA and possible involvement of inhibitory neurons in the VTA.

Notably, activation of the LPB-VTA pathway promoted positive reinforcement in the operant task (Figure 1) whereas it induced avoidance behavior in the place conditioning task in the same individual within the avoider group (Figure 2). Therefore, an intriguing possibility is that the LPB-VTA pathway is involved in positive and negative valence modulation based on the experimental tasks (such as lever-press vs. place conditioning) in a state-dependent manner.

Furthermore, this study showed that opposite behavioral patterns (avoidance and attraction) were detected in the real-time place avoidance test when the LPB-VTA pathway was photoactivated. Although we checked the location of LED cannula implantation, no remarkable bias was observed between avoider and attracted mice (Supplementary Figure S1A). One possible explanation for this opposite behavior is the local circuit of the VTA. In addition to projections from LPB neurons to VTA dopaminergic neurons (Yang et al., 2021), we showed that LPB neurons also project to GAD65 cells (Figure 4). Therefore, LPB projects to both dopamine neurons and inhibitory cells in the VTA. The bias in the types of postsynaptic cells projected by the LPB may result in the opposite behavioral output.

Another possibility for the opposite behavior is the cell type of the LPB neurons. Although the analyses in this study were not limited to a particular LPB cell type, there is a variety of cell types in the LPB. Thus, slight differences in experimental spatial distribution of Chronos-expressing LPB neurons may be connected to the classification of the two groups. Accordingly, it is necessary to elucidate the presynaptic mechanism using pathway-specific or cell-type specific approach in the future investigations. Neurons expressing CGRP and tachykinin-1 (Tac1) in the LPB transmit aversive signals (Han et al., 2015; Barik et al., 2018; Bowen et al., 2020), and pituitary adenylate cyclase-activating polypeptide (PACAP)-expressing neurons projecting from the LPB to BNST enhance anxiety-like behavior (Boucher et al., 2022). In contrast, SatB2 neurons in the PB encode a positive valence that induces appetitive lick behavior (Fu et al., 2019). Sweet and bitter taste stimuli activate LPB neurons projecting to the VTA (Boughter et al., 2019), suggesting that both appetitive and aversive information is transmitted by the LPB-VTA pathway. These cell types with conflicting functions, based on genetic profiles or responses to stimuli, may be involved in the behaviors of avoider and attracted mice observed in the present study. Moreover, Yang et al. demonstrated that the photoactivation of the cell bodies of LPB neurons projecting to VTA dopamine neurons promotes place-preference behavior (Yang et al., 2021). Therefore, the attraction towards the LED area observed in attracted mice can be attributed to the specific LPB cell types projecting to the VTA dopamine neurons.

Activation of the LPB-VTA pathway facilitated positive reinforcement in the lever-press paradigm, although it induced avoidance behavior (Figures 1–3). This seemingly contradicting results may attributable to the differential functions of the LPB-VTA pathway based on the behavioral tasks such as the operant conditioning task, which dependents on voluntary behaviors such as self-administration, and the classical conditioning task, which dependents of involuntary behaviors. To support this notion, brain activity that accompanies free and forced actions is different in humans (Kostelecki et al., 2012), suggesting that the same sensory stimulus could lead to different valences when chosen voluntarily versus involuntarily. Intriguingly, Malenka’ groups demonstrated that activation of the amygdala-nigra pathway in mice elicited reinforcement when linked to voluntary actions, but failed to support Pavlovian associations that rely on incentive value signals (Steinberg et al., 2020). Furthermore, a previous study demonstrated that VTA dopaminergic neuron subpopulations projecting to different regions of the striatum mediate the acquisition of incentive value in Pavlovian associations (Saunders et al., 2018). Collectively, the contribution of this study to the field of neural circuits is that discrete roles of the LPB-VTA based on the behavioral paradigm. Our results indicate that the LPB-VTA pathway could function differently under voluntary action in operant task or involuntarily action in the classical conditioning task while those tasks were performed separately in most previous studies. Understanding how the LPB-VTA pathway is involved in voluntary and involuntary behaviors is an important area for future investigation.

Additionally, some sensory stimuli have been described for their ability to support operant responses as reinforcers. For instance, Winder’s group described a method for assessing non-drug reinforcement using sensory stimuli in mice, termed operant sensation seeking (Olsen and Winder, 2009, 2010). Therefore, another intriguing possibility is that even if the signals transmitted by the LPB-VTA pathway include aversive signals, they may act as reinforcers in the lever-press paradigm through the effect of sensation seeking.

The LPB transmits noxious sensory information to multiple downstream targets, such as the VTA, BNST, CeA, IC, LH, PAG, PSTN, SI, VMH, and VPMpc (Chiang et al., 2019; Bowen et al., 2020; Yang et al., 2021; Nagashima et al., 2022). Recent studies by Palmiter’s group and Ross’s group demonstrated that activation of the cell bodies of LPB neurons or their axon terminals induces defensive behaviors and memory formation (Bowen et al., 2020; Chiang et al., 2020). We have also shown that photoactivation of the LPB-CeA and LPB-PSTN pathways induces avoidance behavior, escape behavior, and aversive memory formation (Ito et al., 2021; Nagashima et al., 2022). Since photoactivation of the LPB-VTA pathway induces avoidance behavior and aversive memory formation in avoider, similar mechanisms may exist in other LPB-dependent pathways. However, avoidance behavior induced by the LPB-PSTN pathway was also accompanied by strong escape behavior (Nagashima et al., 2022), whereas no significant escape behavior was observed in the LPB-VTA pathway (Supplementary Figure S2), suggesting that there are functional differences between the LPB-VTA and LPB-PSTN pathways in the regulation of escape behavior. Moreover, the facilitation of the lever press (Figure 1) and attraction to the LED area (Figure 2) may be specific to the LPB-VTA pathway. Compared to other projection targets of the LPB, dopamine neurons are particularly abundant in the VTA. In fact, dopamine neurons can promote lever-press and attraction behaviors (Yang et al., 2021). Therefore, the unique behaviors induced by photoactivation of the LPB-VTA pathway may be dependent on the activity of dopamine neurons, either through direct or indirect control via inhibitory cells in the VTA.

We photoactivated the axon terminals in the LPB-VTA pathway. Since LPB neurons send collateral projections to various targets, the possibility of antidromic activation cannot be completely ruled out. However, recent studies suggest that terminal stimulation does not produce robust antidromic activation (Bowen et al., 2020; Chiang et al., 2020). In addition, although activation of the LPB-PSTN pathway suppresses lever press (Nagashima et al., 2022), we found that activation of the LPB-VTA pathway promotes lever press. Collectively, terminal stimulation of the LPB-VTA pathway did not appear to produce robust antidromic activation.

Furthermore, we found that pharmacogenetic inhibition of GAD65 cells in the VTA promotes avoidance behavior induced by the photoactivation of the LPB-VTA pathway (Figure 5). A previous study has shown that VTA glutamate neurons promote escape responses to threatening stimuli based on inputs from the lateral hypothalamic area (Barbano et al., 2020). The activity of VTA dopamine and inhibitory neurons can be modulated by GABAergic tonic inhibition (Yang et al., 2023). In the context of regulating avoidance behavior via the LPB-VTA pathway, disinhibition of GAD65-dependent microcircuits in the VTA may activate the dopamine and glutamate neurons involved in defensive behavior.

We manipulated VTA GABAergic neurons using the mGAD promoter (Figure 5). The specificity of the GAD65 mini-promoter for targeting GABAergic neurons was validated through systemic AAV-based expression (Hoshino et al., 2021). However, expression of Chronos may be strongly induced even in cells with low intrinsic promoter activities of GAD65 because of the heightened sensitivity of the Cre-loxP-based approach used in the present study.

Our study suggests that the LPB-VTA pathway can transform conflicting behavioral patterns, such as avoidance and attraction, modulated by GABAergic signaling. These findings advance the current understanding of the neural circuits involved in state-dependent behavioral control. Moreover, sex differences influence the characteristics of the projection from VTA neurons (Manion et al., 2022). In the future, it would be interesting to explore the upstream and downstream circuit mechanisms of the LPB-VTA pathway in both female and male mice. The VTA is implicated in motivated behaviors, addiction, and psychiatric disorders. Further investigation of downstream circuits and molecular mechanisms is crucial for the future discovery and development of new therapies for neuropsychiatric disorders.

## Data availability statement

The original contributions presented in the study are included in the article/Supplementary material, further inquiries can be directed to the corresponding author.

## Ethics statement

The animal study was approved by Institutional Animal Care and Use Committee of the Jikei University (Tokyo, Japan; Approval No. 2018-030 and 2019-010). The study was conducted in accordance with the local legislation and institutional requirements.

## Author contributions

TN: Funding acquisition, Resources, Writing – original draft, Writing – review & editing, Data curation, Formal analysis, Validation. KM: Data curation, Formal analysis, Resources, Writing – review & editing, Methodology. ST: Data curation, Formal analysis, Methodology, Resources, Writing – review & editing. AK: Resources. HH: Resources. AMW: Methodology, Resources, Validation, Writing – review & editing, Conceptualization, Funding acquisition, Investigation, Project administration, Supervision, Visualization, Writing – original draft.

## Footnotes

^1^ http://atlas.brain-map.org/
