# Supplementary material for: State-dependent modulation of positive and negative affective valences by a parabrachial nucleus-to-ventral tegmental area pathway in mice
Source: Front Neural Circuits. 2023 Nov 29;17:1273322. doi: 10.3389/fncir.2023.1273322 (PMC10716301; doi:10.3389/fncir.2023.1273322)
Supplement: Supplementary file 1 [file Image_1.pdf]

Supplementary Figure 1

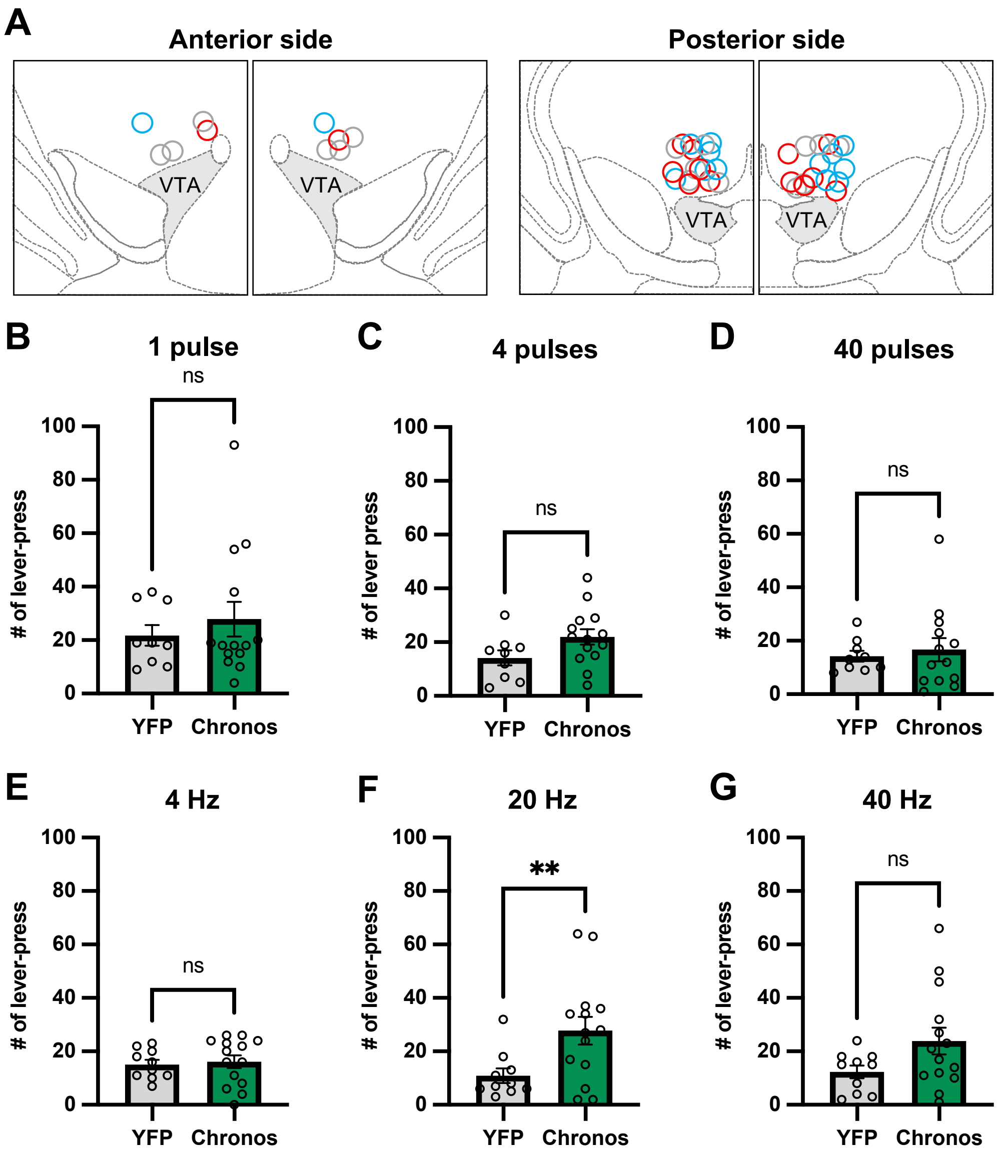

Supplementary Figure 1.
Photoactivation of the lPB-VTA pathway. **(A)** Schematic illustration of the VTA and the approximate positions of the LED cannula tips (gray, YFP; red, Avoider; light blue, Attracted). **(B–D)** Relationship between the number of light pulses and number of lever presses during each 10-min session (YFP, n = 9; Chronos, n = 13–14). **(E–G)** Relationship between stimulus frequency and the number of lever presses during each 10-min session (YFP, n = 10; Chronos, n = 14). ns, $p > 0.05$; $^{**}p < 0.01$ (unpaired two-sided *t*-test). Data are presented as mean ± SEM.

Supplementary Figure 2

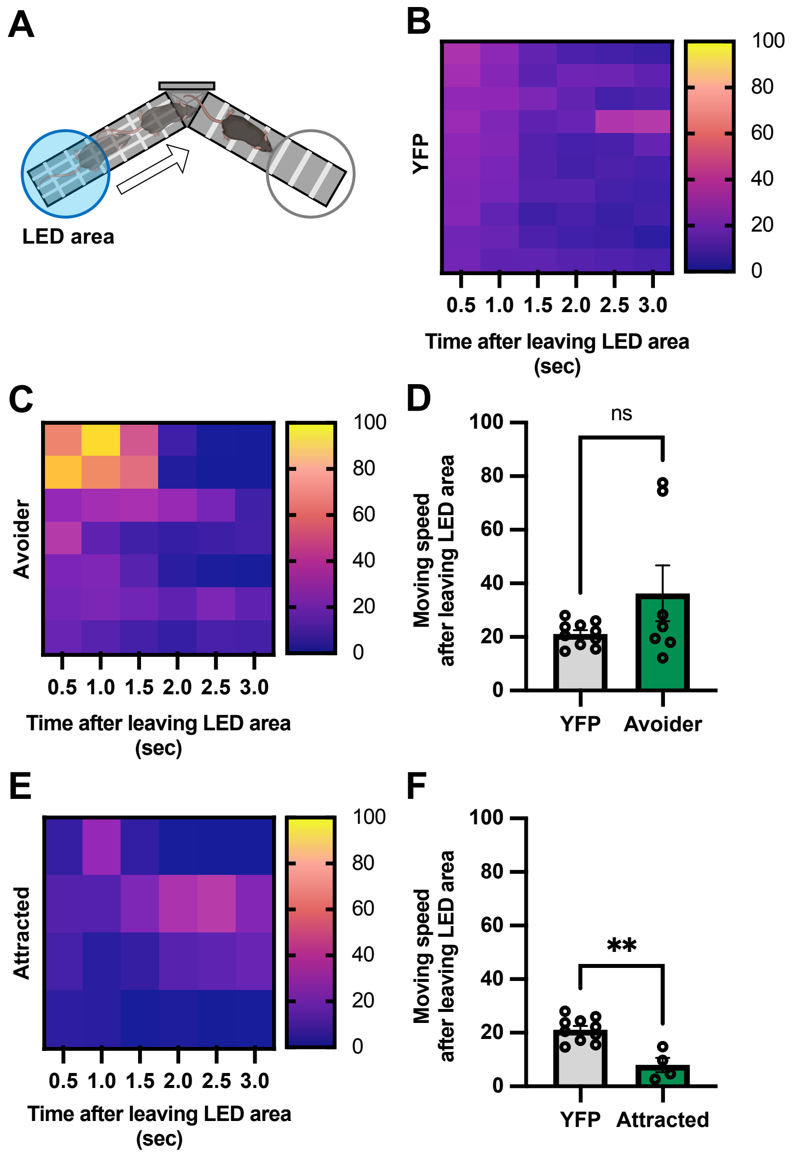

Supplementary Figure 2.
Effects of photoactivation of the lPB-VTA pathway on escape behavior. **(A)** Schematic representation of escape behavior. **(B–F)** Escape behavior of individual mouse. Moving speed for 3 s after leaving the LED area was analyzed at 0.5-s intervals and visualized as a heatmap, where bright colors indicate more rapid movement **(B, C, E)**. Average speed for a second after mice left the LED area during the 10-min conditioning session (YFP, n = 10; Avoider, n = 7; Attracted, n = 4) **(D, F)**. ns, $p > 0.05$; $^{**}p < 0.01$ (unpaired two-sided *t*-test). Data are presented as mean ± SEM.

Supplementary Figure 3

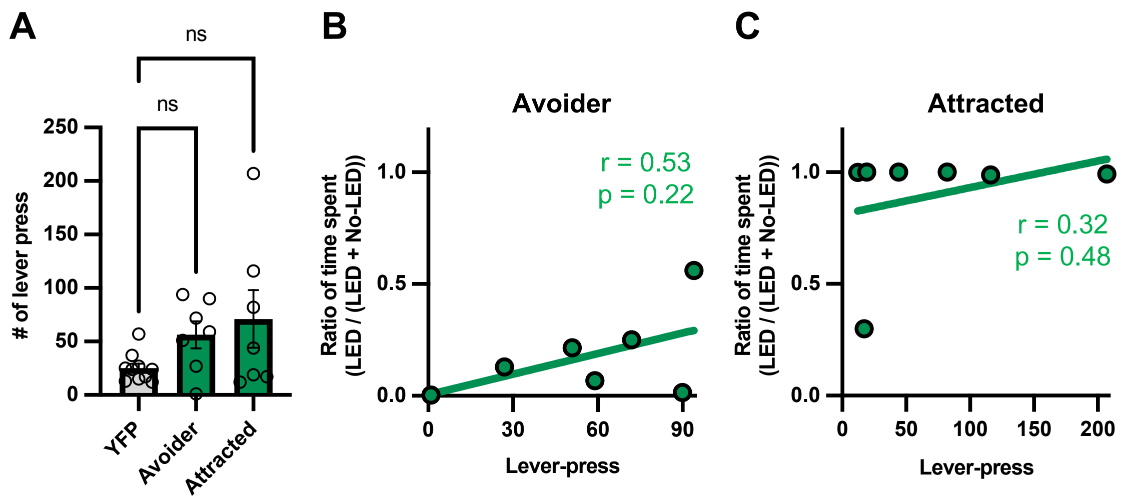

Supplementary Figure 3.
Relationship between lever-press and avoidance behaviors. **(A)** Summary of the number of lever-press responses (YFP, n = 10; Avoider, n = 7; Attracted, n = 7). ns, $p > 0.05$ (one-way ANOVA). Data are presented as mean ± SEM. **(B, C)** Correlations of the number of lever presses and ratio of time spent in each area in the 10-min conditioning session of the real-time place avoidance test. Each dot represents a value obtained from one mouse (Avoider, n = 7; Attracted, n = 7). Pearson's correlation coefficients are shown (Avoider, r = 0.53, $p = 0.22$; Attracted, r = 0.32, $p = 0.48$).

Supplementary Figure 4

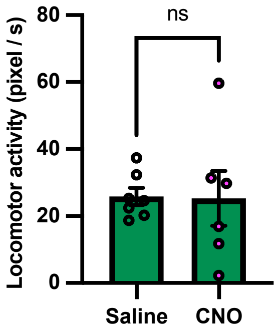

Supplementary Figure 4.
The effect of chemogenetic inhibition of GAD65 cells on locomotor activity. Locomotor activity before optogenetic stimulation (Saline, n = 7; CNO, n = 6). Animals that entered the LED-area within 1 s after the start of the session were excluded. ns, $p > 0.05$ (unpaired two-sided t-test). Data are presented as mean ± SEM.
